# Sonographic Pattern of Subacute Thyroiditis Is HLA-Dependent

**DOI:** 10.3389/fendo.2019.00003

**Published:** 2019-01-23

**Authors:** Magdalena Stasiak, Bogusław Tymoniuk, Zbigniew Adamczewski, Bartłomiej Stasiak, Andrzej Lewiński

**Affiliations:** ^1^Department of Endocrinology and Metabolic Diseases, Polish Mother's Memorial Hospital Research Institute, Lodz, Poland; ^2^Department of Immunology, Rheumatology and Allergy, Medical University of Lodz, Lodz, Poland; ^3^Department of Endocrinology and Metabolic Diseases, Medical University of Lodz, Lodz, Poland; ^4^Institute of Information Technology, Lodz University of Technology, Lodz, Poland

**Keywords:** HLA-B^*^35, HLA-B^*^18:01, HLA-DRB1^*^01, HLA-C^*^04:01, subacute thyroiditis, ultrasound

## Abstract

**Background:** Since 1977 the susceptibility to SAT has been known to be HLA-B^*^35-related in ~70% of patients. Recently it has been demonstrated that SAT is associated with the presence of HLA-B^*^18:01 and DRB1^*^01, as well as with HLA-C^*^04:01. The association between the type of genetic SAT background and sonographic pattern of the disease has never been analyzed. The aim of the study was to evaluate the potential correlation between the presence of individual HLA haplotypes and the sonographic SAT pattern, and to provide the US characteristics of the analyzed SAT cases.

**Methods:** HLA-A, -B, -C, -DQB1, and -DRB1 were genotyped using a next-generation sequencing method in 46 SAT patients. All patients were divided into the following groups according to the HLA haplotype: 1. HLA-B^*^35 and/or HLA-C^*^04, but without any other of the analyzed antigens; 2. HLA-DRB1^*^01, regardless of the co-presence of HLA-B^*^35 or C^*^04:01, but without HLA-B^*^18:01; 3. HLA-B^*^18:01 only, without any other of the analyzed antigens; 4. HLA-B^*^18:01 plus B^*^35, regardless of the presence of any other analyzed antigens. The US patterns of SAT thyroid lesions were compared among the groups.

**Results:** The US image of SAT lesions in Groups 1 and 2 were similar. The typical SAT features for these groups were as follows: hypoechoic, strongly heterogeneous, bilateral, multiple areas, with decreased vascularization, usually oval with blurred margins, infrequently affecting the whole lobe, or having nodule-like pattern. Several features of Group 3 were different from the other groups. In 60% of cases lesions were rather homogeneous, and in 100%–hypoechoic, in 80% of patients there was only one unilateral single SAT area filling the whole affected lobe. On the contrary to the other groups, in Group 4 no lesion was oval in shape.

**Conclusions:** Our results provide for the first time the evidence that the US pattern of SAT lesions depends on HLA, and the determining factor is the presence of HLA-B^*^18:01. The deviations from the typical SAT US image are mostly pronounced in patients with the presence of only HLA-B^*^18:01, without any other analyzed haplotype. Further research is necessary to explain this phenomenon.

## Introduction

Subacute thyroiditis (SAT) (also called de Quervain thyroiditis) is a thyroid inflammatory disease, associated with the presence of certain types of human leukocyte antigens (HLA). Previous viral infection (occurring ~2–6 weeks earlier) is considered a triggering factor in genetically predisposed individuals. The prevalence of SAT is the highest in middle-aged women, and male to female ratio varies from 1:4 (1) to 1:7 (2). The most common presentation is the anterior neck pain radiating ipsilaterally up to the jaw and ear, and down to the upper part of the chest (3, 4). Fever is usually present, frequently reaching over 39°C, and rising especially at night. Patients often complain of asthenia, and some symptoms of thyrotoxicosis are often present. Typical laboratory findings include high erythrocyte sedimentation rate (ESR). The level of C reactive protein (CRP) is also elevated and laboratory markers of hyperthyroidism are often present but the blood concentration of thyroid antibodies is normal in most patients (3, 4).

Sonographic findings in SAT can be very diverse. In most cases, the ultrasound (US) features include hypoechoic and heterogeneous areas, poorly vascularized on color Doppler, usually with blurred margins and without microcalcifications (5, 6). High tissue stiffness in sonoelastography is characteristic for the disease (7). Blurred, heterogeneous areas of different shape and size can be described as “lava-flow” sonographic pattern (5). The SAT lesions can be bilateral or unilateral, sometimes switching the affected side in the course of the disease. However, the US pattern of SAT is sometimes non-characteristic, and SAT-associated thyroid lesions may mimic thyroid nodules, sometimes with suspicious US features (6).

Since 1977 the susceptibility to SAT has been known to be HLA-B^*^35-related in ~70% of patients (8–10). Recently, our research group demonstrated that SAT is associated with the presence of HLA-B^*^18:01 and DRB1^*^01, as well as with HLA-C^*^04:01, with the latter being in linkage disequilibrium with HLA-B^*^35 (Stasiak et al., under review). Haplotypes HLA-B^*^18:01 and DRB1^*^01 are HLA-B^*^35-independent SAT risk factors (Stasiak et al., under review). These four (4) antigens allow confirmation of the genetic basis in almost all patients with SAT. However, the association between the type of genetic SAT background and sonographic pattern of the disease has never been analyzed.

The aim of the study was to evaluate the potential correlation between the presence of SAT-associated HLA haplotypes, i.e., HLA-B^*^18:01, DRB1^*^01, B^*^35, and C^*^04:01, and the sonographic SAT pattern, and to provide the US characteristics of the analyzed SAT cases.

## Materials and Methods

High resolution HLA typing was performed in 46 patients who were diagnosed with SAT between 2003 and 2018 in the Department of Endocrinology and Metabolic Diseases, Polish Mother's Memorial Hospital—Research Institute, Lodz, Poland. HLA-A, -B, -C, -DQB1, and -DRB1 were genotyped using a next-generation sequencing method on Illumina platform (Illumina, USA). Sequencing-based HLA typing of the HLA genes A, B, C, DQB1, and DRB1 was carried out in 96-well format within a semi-automated workflow by using MiaFora Flex5 typing kits (Immucor, USA). Long-range PCR amplification of five HLA loci was performed on DNA extracted from blood samples.

The diagnosis of SAT was based on the diagnostic criteria recently proposed by our research group (2). These criteria were as follows: elevation of ESR (or at least CRP) plus hypoechoic area/areas with blurred margin and decreased vascularization in US, plus cytological confirmation of SAT (or at least cytological exclusion of malignancy), plus at least one of the following: hard thyroid swelling and/or pain and tenderness of the thyroid gland/lobe, and/or elevation of serum free thyroxine (FT4) and suppression of thyrotropin (TSH), and/or decreased radioiodine uptake (RAIU). Serum levels of TSH and FT4 were measured by electrochemiluminescence immunoassay (ECLIA), Cobas e601 analyzer (Roche Diagnostics, USA), ESR was determined with Ves-Matic Cube 30 (Diesse, Italy), CRP was determined by VITROS® 4600 Chemistry System (Ortho Clinical Diagnostics, USA).

Ultrasound examination (US) was performed in every patient, in a supine position, using a Toshiba Aplio XG scanner (Toshiba, Japan), with high frequency 7–14 MHz linear transducer with field of view width of 38 mm (PLT 1204 BT). The US examinations were performed with standard settings optimized for thyroid imaging. The protocol included the B-mode presentation with application of image improvement techniques such as spatial compound imaging and differential tissue harmonics. In Power Doppler US, a pulse repetition frequency (PRF) 14.1 KHz and color gain 60 were routinely used for evaluating thyroid lesions vascularity. The Power Doppler gain was controlled so that perithyroidal tissues did not display any random color artifacts. The mean size of SAT lesions was calculated on the basis of the largest of the three dimensions of the analyzed lesions. The analyzed US features were defined as follows: 1. echogenicity: moderately hypoechoic were lesions darker than the normal surrounding thyroid parenchyma, but less dark than the surrounding muscles; markedly hypoechoic were lesions at least as dark as the surrounding strap muscles; 2. strongly heterogeneous were lesions with mixed isoechoic, hypoechoic and strongly hypoechoic echogenicity; 3. microcalcifications were calcifications smaller than 1 mm; 4. shape: lesion was considered as oval if its anteroposterior diameter was shorter than the transverse diameter on both transverse and longitudinal planes; lesion was considered as round if its anteroposterior and transverse diameters were equal on both transverse and longitudinal planes; lesion was considered as patchy if its shape was entirely irregular and could not be classified as oval or round; 5. margins: margins were considered as blurred if there was no clear separation from the surrounding thyroid parenchyma; margins were considered as lobulated if at least one oval or round protrusion of the margin was present; margins were considered as smooth if they were clearly separated from the surrounding thyroid parenchyma; 6. the lesions were considered as nodule-like if they resembled thyroid nodules but disappeared with SAT resolution and were previously cytologically confirmed as SAT; 7. the areas were considered as blurred if their shape and margins were difficult to determine; the areas were considered as separated if their shape and margins could be clearly determined, even if the shape was irregular. Vascularity was determined with Power Doppler. The following definitions for vascularity were used: 1. decreased—blood flow within the lesion present but decreased as compared to the normal thyroid parenchyma; 2. normal or increased—blood flow within the lesion present with the same or increased intensity as compared to the normal thyroid parenchyma; 3. absent—no flow detected within the lesion.

Fine needle aspiration biopsy (FNAB) was performed in all SAT patients using a 23-gauge needle. For cytology, hematoxylin-eosin staining was used for evaluation. The smear was considered as diagnostic if at least five groups of well-preserved follicular cells, containing at least 10 cells each, were visible. Smears were cytologically evaluated, and the presence of multinucleated giant cells together with mononucleated macrophages, and follicular epithelial cells against acute and chronic inflammatory dirty background was considered as a result typical for SAT. Cytological confirmation of microcalcification was possible if characteristic violet calcium salt deposit was visible in hematoxylin-eosin stained FNAB material.

All patients were divided into the following groups according to the HLA haplotype: 1. HLA-B^*^35 and/or HLA-C^*^04, but without any other of the analyzed antigens; 2. HLA-DRB1^*^01, regardless of the co-presence of HLA-B^*^35 or C^*^04:01, but without HLA-B^*^18:01; 3. HLA-B^*^18:01 only, without any other of the analyzed antigens; 4. HLA-B^*^18:01 plus B^*^35, regardless of the presence of any other analyzed antigens.

For each HLA group the difference in US pattern of SAT thyroid lesions between this group and all the remaining cases was analyzed. Additionally, a pair-wise comparison was also done for every possible pair of groups. Statistical analysis of several US SAT features was performed for each group individually. The respective variables were mostly categorical, usually concerning the presence/absence of a particular feature. SAT lesion size was the only numerical parameter, for which descriptive statistics, including mean, standard deviation, minimum and maximum values, were computed in each group. Student's *t*-test was applied to determine the statistical significance of the differences between groups, while for the categorical variables chi-square test was used for this purpose. In all the tests the value of *p* < 0.05 was considered significant.

Written informed consents for all the performed procedures were obtained from all the patients.

The study was approved by Local Medical Ethics Committee.

## Results

### General Sonographic Characteristics

Hypoechogenicity of SAT lesions was characteristic for all the cases, and in 5 patients (11%) SAT lesions were deeply hypoechoic. Among 46 patients, in 36 (78.3%) the SAT lesions were strongly heterogeneous, and in 10 (21.7%) they were rather homogeneous in their hypoechogenicity. Microcalcifications (confirmed cytologically) were present in one case only (2.2%). The mean size of SAT lesions was 38.2 mm (ranging from 9 to 70 mm). In 27 patients (58.7%) the SAT lesion/lesions completely filled one or both of the thyroid lobes. In patients in whom the SAT lesion did not fill the whole affected lobe, the shapes of the lesions were as follows: oval—in 69.2%, patchy—in 23.1% and round—in 7.7%, while the margins were blurred in 80.8%, lobulated—in 11.5% and smooth—in 7.7%. SAT lesions were bilateral in 76% and unilateral in 24%. The SAT lesions resembled thyroid nodules in 8.7%. Among SAT-typical areas, in 19.6% of the cases there was one isolated area corresponding to tender palpable nodule. In all the remaining cases the areas were multiple. The vascularization of SAT lesions were decreased in 82.6% of the cases, normal or increased in 8.7% and absent in 8.7%.

### HLA-Dependent Sonographic Characteristics

All the evaluated SAT features in the analyzed groups have been presented in Table 1, along with the comparison of every single group with all the other subjects. Comparisons between individual groups have been presented in Table 2.

**Table 1:** Sonographic characteristics of the SAT lesions in each group and comparison with all the other subjects.

**Sonographic feature of SAT lesions**	**Group 1 (*n* = 25)**	*p*-value^*^	**Group 2** **(*n* = 10)**	*p*-value^*^	**Group 3** **(*n* = 5)**	*p*-value^*^	**Group 4** **(*n* = 6)**	*p*-value^*^
Echogenicity		0.788		0.294		0.408		0.359
Moderately hypoechoic	88%		80%		100%		100%	
Markedly hypoechoic	12%		20%		0%		0%	
Strongly heterogeneous	84%	0.303	80%	0.880	40%	**0.028**	83.3%	0.747
Microcalcifications	4%	0.353	0%	0.594	0%	0.724	0%	0.695
Shape		0.909		0.785		0.656		**0.027**
Oval	73.3%		66.7%		100%		0%	
Patchy	20%		33.3%		0%		50%	
Round	6.7%		0%		0%		50%	
Margin		0.937		0.572		0.602		0.062
Blurred	80%		100%		66.7%		50%	
Lobulated	13.3%		0%		33.3%		0%	
Smooth	6.7%		0%		0%		50%	
Bilateral	84%	0.170	70%	0.610	20%	**0.002**	100%	0.141
Whole lobe affected	48%	0.141	70%	0.606	80%	**0.003**	66.7%	0.777
Nodule-like pattern	8%	0.855	10%	0.869	0%	0.465	16.7%	0.457
Blurred areas	28%	0.747	40%	0.257	0%	0.159	16.7%	0.573
Separated areas	36%	0.592	30%	0.842	60%	0.166	0%	0.068
No. of areas		0.506						
Single area	16%		10%	0.389	80%	**<0.0005**	0%	0.195
Multiple areas	84%		90%		20%		100%	
Vascularity								
Decreased	84%	0.786	90%	0.486	60%	0.431	83.3%	0.960
Normal or increased	12%	0.385	0%	0.270	0%	0.915	16.7%	0.457
Absent	4%	0.217	10%	0.869	40%	0.073	0.0%	0.418

* *As compared to all other groups*.

*p < 0.05 was considered as statistically significant*.

Group 1. HLA-B^*^35 and/or HLA-C^*^04, but without any other of the analyzed antigens;

Group 2. HLA-DRB1^*^01, regardless of the co-presence of HLA-B^*^35 or C^*^04:01, but without B^*^18:01;

Group 3. HLA-B^*^18:01 only, without any other analyzed antigen;

Group 4. HLA-B^*^35 plus B^*^18, regardless of the presence of any other analyzed antigens.

**Table 2:** Comparison of sonographic features of the SAT lesions in pairs of the analyzed groups.

**Sonographic feature**	***p*-value for groups 1 and 2**	***p*-value for groups 1 and 3**	***p*-value for groups 1 and 4**	***p*-value for groups 2 and 3**	***p*-value for group 2 and 4**	***p*-value for** **groups 3 and 4**
Echogenicity	0.541	0.414	0.372	0.283	0.242	1.000
Strongly heterogenous	0.777	**0.034**	0.968	0.121	0.869	0.137
Microcalcifications	0.521	0.649	0.618	1.000	1.000	1.000
Shape	0.688	0.598	0.084	0.257	0.108	0.082
Margin	0.497	0.651	0.193	0.134	0.064	0.329
Bilateral	0.350	**0.003**	0.294	0.067	0.137	**0.006**
Whole lobe affected	0.490	**0.004**	0.714	0.060	0.982	0.079
Nodule-like pattern	0.849	0.513	0.519	0.464	0.696	0.338
Blurred areas	0.490	0.177	0.569	0.099	0.330	0.338
Separated areas	0.735	0.317	0.081	0.264	0.137	**0.026**
Single/multiple areas	0.647	**0.003**	0.294	**0.007**	0.424	**0.006**
Vascularity						
Decreased	0.647	0.221	0.968	0.171	0.696	0.387
Normal	0.252	0.414	0.759	1.000	0.182	0.338
Absent	0.490	**0.014**	0.618	0.171	0.424	0.087

*p < 0.05 was considered as statistically significant*.

Group 1. HLA-B*35 and/or HLA-C*04, but without any other of the analyzed antigens;

Group 2. HLA-DRB1*01, regardless of the co-presence of HLA-B*35 or C*04:01, but without B^*^18:01;

Group 3. HLA-B*18:01 only, without any other analyzed antigen;

Group 4. HLA-B*35 plus B*18, regardless of the presence of any other analyzed antigens.

**Figure 1:**
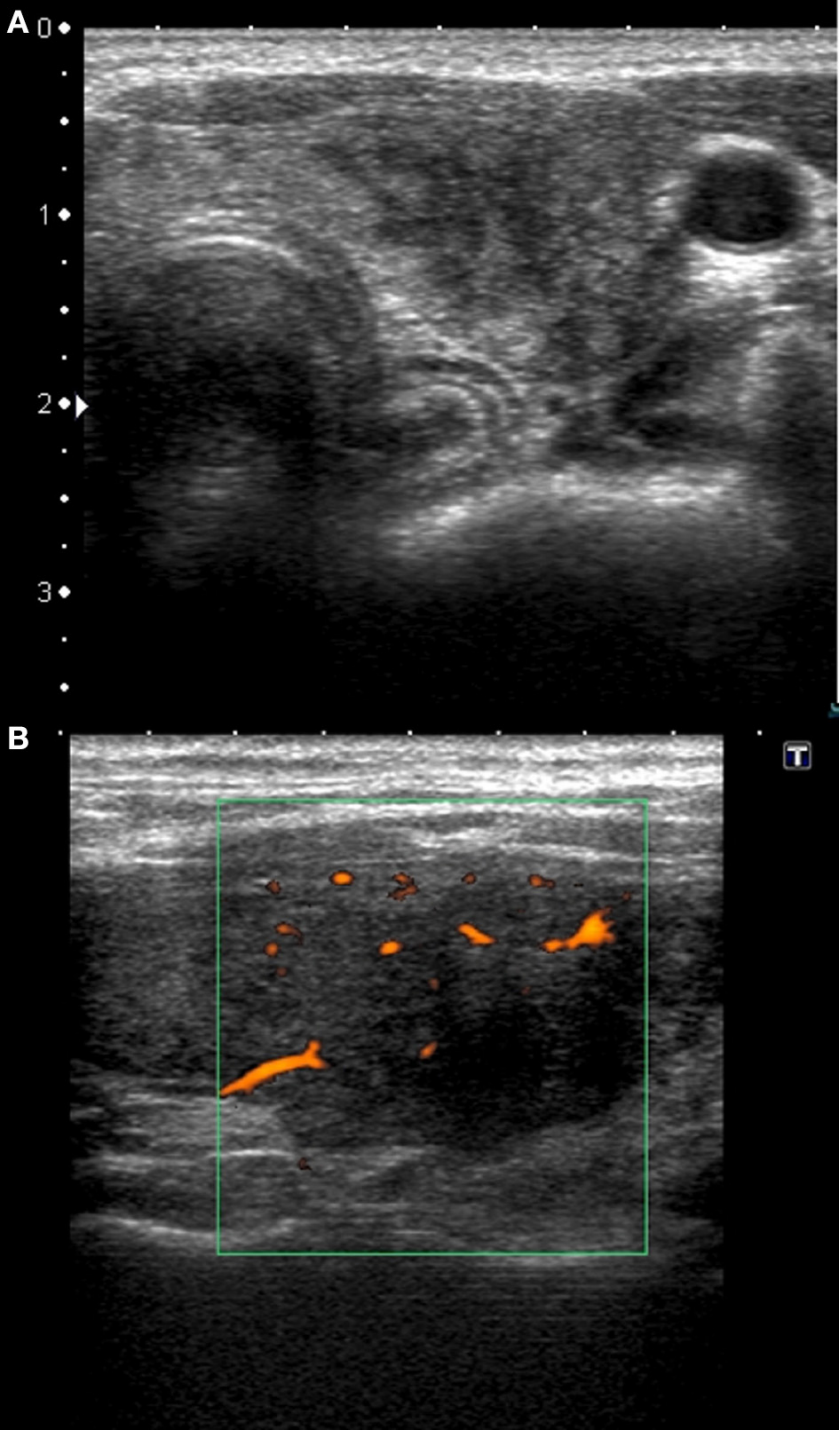
Typical sonographic patterns of SAT in patients with HLA-B*35, C*04:01, and DRB1*01, without HLA-B*18:01. **(A)** Hypoechoic, strongly heterogeneous, areas with poorly defined margins; **(B)** hypoechoic blurred areas with decreased or absent vascularization.

The US images of SAT lesions in Groups 1 and 2 were similar (Table 1), with no statistically significant differences between these two Groups (Table 2). The typical SAT features for these Groups were as follows: hypoechoic (88% in Group 1 vs. 80% in Group 2), strongly heterogeneous (84 vs. 80%), bilateral (84 vs. 70%), multiple (84 vs. 90%) areas, with decreased vascularization (84 vs. 90%), usually oval (73.3 vs. 66.7%) with blurred margins (80 vs. 100%), infrequently affecting the whole lobe (48 vs. 70%), rarely with nodule-like pattern (8 vs. 10%) (Figure 1).

**Figure 2:**
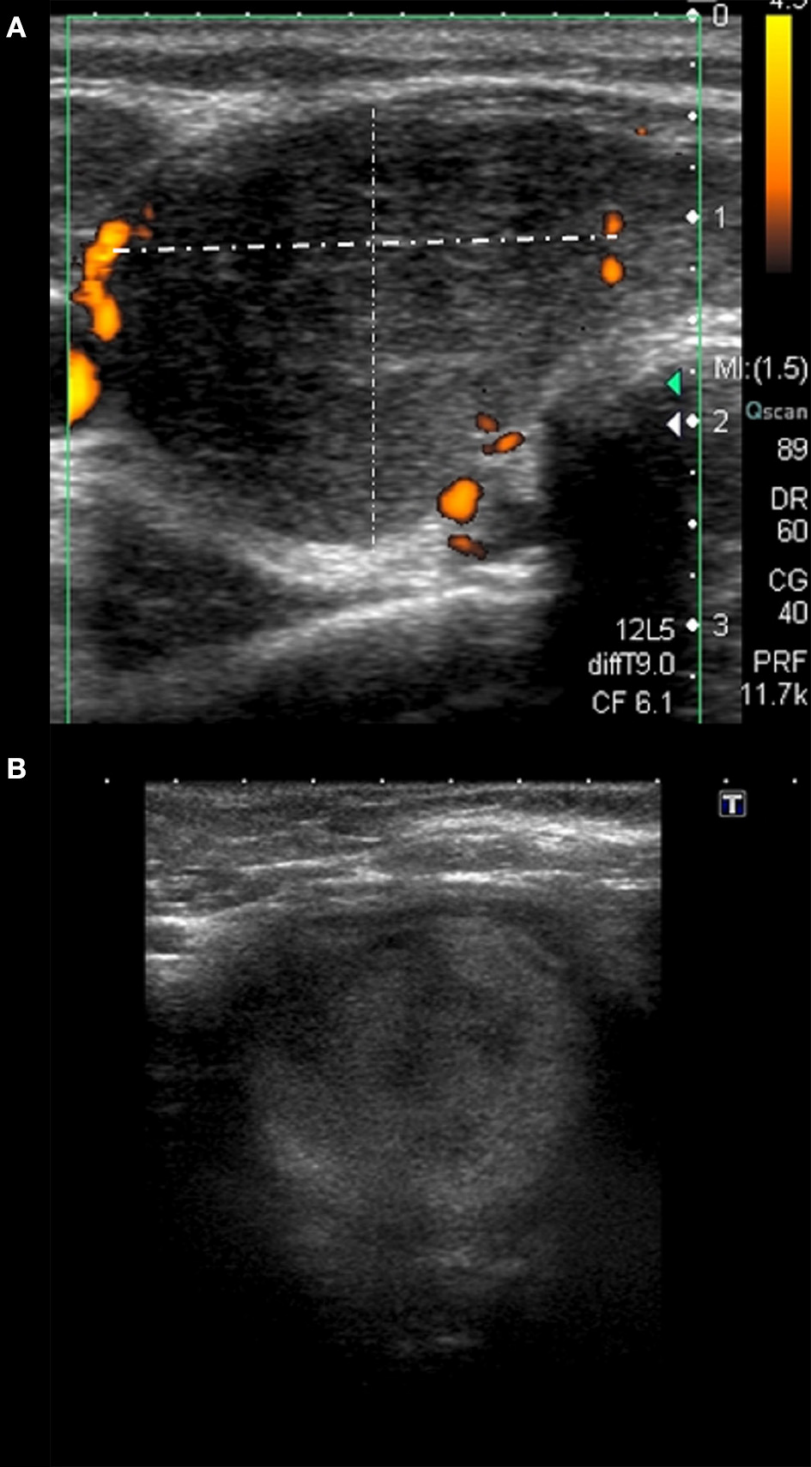
Different sonographic pattern observed in patient with HLA-B*18:01 only. **(A)** Homogeneously hypoechoic single area filling the whole right lobe; **(B)** moderately hypoechoic single SAT area filling the whole thyroid lobe, mimicking a nodule affecting the whole lobe.

Several features of Group 3 were different from the other groups (Tables 1, 2). In only 40% of the cases, lesions were strongly heterogeneous (*p* = 0.028 as compared to the other groups), while in 60% they were rather homogenous, and in 100% hypoechoic. Only 20% of these patients had bilateral lesions (*p* = 0.002 as compared to other groups), but in 80% of them there was only one single SAT area (*p* < 0.0005 as compared to other groups), filling the whole affected lobe (*p* = 0.003, as compared to other groups) (Figure 2). In Group 3, the vascularization was absent in as much as 40% of patients, although the difference was statistically significant only in comparison with Group 1 (*p* = 0.014). Other US findings were similar as in the other groups (Tables 1, 2). In contrast to all other groups, in Group 4 no lesion was oval in shape (*p* = 0.027 as compared to other groups). Other differences were not statistically significant (Tables 1, 2).

In comparison of both HLA-B^*^18:01-positive Groups (3 and 4), significant differences were observed in lateralization, as 80% of the patients in Group 3 had unilateral lesions, while 100% of the patients in Group 4 had bilateral lesions (*p* = 0.006) (Table 2). Moreover, single (80%), separated (60%) areas were typical for Group 3, while these features were absent in Group 4 (*p* = 0.006 and *p* = 0.026, respectively) (Table 2).

There were no statistically significant differences in the SAT lesion size between the groups (Table 3).

**Table 3:** Size of the SAT lesions in the analyzed group of patients.

	**Mean (mm)**	**SD**	**Min (mm)**	**Max (mm)**	*p*-value^*^
Group 1	38.13	16.94	9.0	70.0	0.934
Group 2	34.10	15.99	13.0	62.0	0.361
Group 3	42.40	15.66	25.0	66.0	0.564
Group 4	42.67	14.84	19.0	63.0	0.471

*As compared to all other groups.

*p < 0.05 was considered as statistically significant*.

Group 1. HLA-B*35 and/or of HLA-C*04, but without any other of the analyzed antigens;

Group 2. HLA-DRB1*01, regardless of the co-presence of HLA-B*35 or C*04:01, but without B^*^18:01;

Group 3. HLA-B*18:01 only, without any other analyzed antigen;

Group 4. HLA-B*35 plus B*18, regardless of the presence of any other analyzed antigens.

*max, maximum size; min, minimum size; SD, standard deviation*.

## Discussion

Ultrasound evaluation plays an important role in the diagnostic process and in monitoring of SAT. Clinical SAT symptoms may be non-characteristic and US examination helps to make the proper diagnosis. The typical sonographic findings of SAT are bilateral or unilateral, focal or multifocal, poorly defined hypoechoic areas (5, 6, 11, 12) in the enlarged thyroid gland. In some cases, nodular lesions can be observed, sometimes mimicking thyroid malignancy (13, 14). SAT lesions were typically hypoechoic in all our cases, and in 5 patients (11%) they were even deeply (markedly) hypoechoic. Lee and Kim (11) observed markedly hypoechoic lesions only in patients with the nodule-like US pattern of SAT. On the contrary, in our group, all markedly hypoechoic lesions were blurred areas, while all nodule-like lesions were moderately hypoechoic. These discrepancies are most probably the result of different criteria of lesion qualification as nodule-like one, since Lee and Kim described nodular US pattern of SAT as more frequent than non-nodular (11). In our study, SAT lesions resembled thyroid nodules only in 8.7%, and all the remaining lesions were non-nodular areas. Similarly to other authors' observations (5, 6, 12), most of the SAT lesions (78.3%) were strongly heterogeneous and microcalcifications were an extremely rare finding.

The mean size of the SAT lesions was 38.2 mm (ranging from 9 to 70 mm). Similar size range was observed by Vural et al. (6) who described lesions sized 7–75 mm in a group of 20 SAT patients, reporting also that the largest lesions filled the whole thyroid lobe, sometimes even together with isthmus. In our cohort, 58.7% of the patients had lesions that completely filled one or both thyroid lobes. In patients, in whom the SAT lesion did not fill the whole affected lobe, the lesions had various shapes. In our study, oval lesions were the most common type, occurring in 69.2%, while patchy, and round lesions constituted 23.1 and 7.7%, respectively. Lee and Kim (11) reported all non-nodular lesions as patchy in shape, and round areas were not observed.

Bilateral occurrence of SAT lesions is common, usually exceeding 70% of patients (5, 11, 12) and in our study the prevalence of bilateral SAT lesions was 76%. Interestingly, Park et al. (15) demonstrated the presence of bilateral SAT lesions only in 14.8% of patients. This discrepancy in the scale of thyroid involvement, as compared to our observations, may result from the lower SAT severity in the study group analyzed by Park et al. (15), as compared to ours. This conclusion seems to be supported by the fact that the ESR in the group of Park et al. (15) was only slightly elevated, with a mean value of 37 mm/hr (normal range 0–20), and in 25% of the patients the ESR was even normal, which can question SAT diagnosis in the cases without elevated ESR. For comparison, in our group, all the patients had elevated ESR, and the mean value was 66 mm/h, with the upper normal limit of 12 mm/h (data not presented in this paper).

The vascularization of SAT lesions is usually decreased (82.6% of our cases) or even absent (8.7% of our cases) although several authors reported normal to increases blood flow in a few SAT lesions, as well (11, 12, 16). Prior studies suggested that decreased vascularization is typical for the acute SAT phase and the increased blood flow is a feature of the recovery phase (12, 16). However, this explanation cannot be applied to our patients, because the analyzed US image was the first one, performed to diagnose the disease in the acute phase (confirmed by increased inflammation markers, including markedly increased ESR in all the cases), and yet we observed increased or normal vascularization in 8.7% of patients.

The occurrence of SAT is associated with genetic predisposition, encoded in the sixth chromosome fragment responsible for the HLA tissue compatibility antigen system. Correlation between SAT and HLA-B^*^35 has widely been described (8, 17–22) but the genetic SAT background in HLA-B^*^35-negative patient was unknown. Recently, the strong association between SAT and HLA-B^*^18:01, DRB1^*^01 and HLA-C^*^04:01 was demonstrated by our study team (Stasiak et al., under review) and—therefore—currently the HLA-dependent background of SAT is possible to be determined in almost all patients.

No data regarding the association of HLA haplotype and US pattern of SAT was available, because until recently, only HLA-B^*^35 was unequivocally considered to be a genetic SAT background. In most studies analyzing this relationship, no US examination was performed, or no description of the sonographic findings was available. The first reports on the relationship of SAT and HLA-B^*^35 date back to the 1970's, when the US examination was not widely available. The authors of the subsequent articles regarding the significance of HLA-B^*^35 in SAT used RAIU in the diagnosis of the disease, or the diagnosis was based only on the clinical presentation, elevated ESR and laboratory features of thyrotoxicosis (8, 17–20, 22). Therefore, only very few thyroid US images are described in SAT patients with confirmed presence of HLA-B^*^35. Hamaguchi et al. (21) reported HLA-B^*^35-positive twins with SAT, in whom the thyroid was enlarged in US, with unilateral hypoechoic areas corresponding to tender, firm, palpable nodules. Unfortunately, no details concerning the size or shape of the areas were provided (21). In our study, HLA-B^*^35 and HLA-C^*^04:01 were analyzed together in the same group, as HLA-C^*^04:01 is in linkage disequilibrium with HLA-B^*^35. The typical sonographic pattern for this group included multiple hypoechoic, strongly heterogeneous, areas, with decreased vascularization, usually oval, with blurred margins, infrequently affecting the whole lobe. In contrast to the observations of Hamaguchi et al. (21), in our Group 1 SAT lesions were often bilateral (84%). It should be stressed, however, that a single description of identical twins cannot be compared to the analysis of our group, containing 25 patients. The US pattern of SAT lesions in our Group 1 corresponds to the typical SAT sonographic findings. Interestingly, the same typical pattern was found in HLA-DRB1^*^01-positive group, although this haplotype belongs to class II of the major histocompatibility complex (MHC), while all other HLA-related SAT risk haplotypes belong to class I MHC. It could have been expected that the main differences in the US image should be found between haplotypes from various MHC classes. However, it seems that the effect of haplotypes HLA-B^*^35, C^*^04:01 and DRB1^*^01 on the US image of SAT lesions is similar.

It is worth noticing that no statistically significant differences in patient age between the analyzed four groups has been found (data not presented in the present paper). Recently Vita et al. (23) reported that among patients with remission of Graves' disease, carriers of the alleles B35 or DR1 (serotyping) were the youngest and the oldest, respectively. However, the peak incidence age is different for each of the two diseases, and therefore discrepancies in age-dependence are expectable.

The presence of HLA-B^*^18:01 seems to be a key factor that changes the thyroid US image in SAT, because the differences in sonographic findings are expressed most strongly in patients with HLA-B^*^18:01 only. Less expressed differences from the typical SAT US pattern are visible in patients with the coexistence of HLA-B^*^18:01 and B^*^35, while in patients without HLA-B^*^18:01 sonographic findings are SAT-typical. Several features of SAT lesions in patients with the presence of HLA-B^*^18:01 only were different from patients without HLA-B^*^18:01 haplotype or even from patients with simultaneous presence of HLA-B^*^18:01 and B^*^35. In most cases with HLA-B^*^18:01 only, there was unilateral homogenously hypoechoic single SAT area, which filled the whole affected lobe, mimicking the large thyroid nodule. Vascularization of these lesions was SAT-typical—decreased or frequently absent. In patients with co-presence of HLA-B^*^18:01 and B^*^35, the main difference, as compared to the other groups, concerned the shape of the SAT lesions. In the whole group of our patients, the most common SAT lesion shape was oval, but no lesion in HLA-B^*^18:01 plus B^*^35 patients was oval in shape. The lesions were patchy or round—the latter shape being very rare in SAT lesions.

Our results have clearly shown that the presence of HLA-B^*^18:01 is the main factor changing the typical SAT sonographic pattern to a different one. The co-occurrence of HLA-B^*^35 reduces many of the above described differences, although the presence of HLA-B^*^18:01 still alters the US pattern, which remains—to some extent—different from the one typical for SAT. It is to be noted that the differences between the US image in HLA-B^*^18:01-positive patients with and without HLA-B^*^35 are very significant. Firstly, in 80% of patients with HLA-B^*^18:01 only, SAT lesions were unilateral, while in patients with additional presence of HLA-B^*^35 all lesions were bilateral. Secondly, in the HLA-B^*^18:01 group, most lesions were single, separated, homogenously hypoechoic areas filling the whole thyroid lobe, while for patients with HLA-B^*^18:01 and B^*^35, multiple heterogeneous areas were a typical US finding. Now, it is difficult, to speculate why the occurrence of HLA-B^*^18:01 changes the US SAT image in such a great extent. Perhaps the decisive role is played by subpopulations of immune cells that are involved in the inflammatory process in SAT. The presence on the cell membrane of MHC class I molecules presenting own peptides that resemble pathogen antigens increases the risk of antigen presentation, effector cell activation and the occurrence of the inflammatory process. Further research is required to precisely explain this phenomenon—first of all, our future work will focus on evaluation of the inflammatory cells subpopulation in the FNAB material obtained in the active phase of SAT inflammation in patients with particular HLA haplotypes.

When analyzing the conclusions drawn on the basis of the obtained results, the limitations of this study should be taken into account. Firstly, it should be noted that all the patients taking part in the study were Caucasians, and the direct transposition of the obtained results to other populations may not be fully possible. Secondly, ultrasound examination was performed by the authors, so it was not blinded for SAT diagnosis. However, it was fully blinded for HLA antigens, because no HLA results were known at the time of US examination in the acute phase of the disease. Moreover, the pathology review was unblinded as the suspicion of SAT was always stated on the cytology request form. Additionally, the analysis of the obtained data must be done with care in regard to the statistical significance, as—despite the relatively large number of the analyzed parameters—no correction for multiple comparisons was applied. However, in most of the cases marked bold in Tables 1, 2 we can assume that the presented *p*-values indicate the true, existing differences between the groups with high probability.

In conclusion, our results provide for the first time evidence that the US image of SAT depends on HLA haplotype, and the determining factor is the presence of HLA-B^*^18:01. The deviations from the typical SAT US image are mostly pronounced in patients with the presence of only HLA-B^*^18:01, without any other analyzed haplotypes. Further research is necessary to explain the cause of this phenomenon.

## Ethics Statement

This study was carried out in accordance with the recommendations of WHO Standards and operational guidance for ethics review of health-related research with human participants with written informed consent from all subjects. All subjects gave written informed consent in accordance with the Declaration of Helsinki. The protocol was approved by the Ethics Committee of the Polish Mother's Memorial Hospital—Research Institute, Lodz, Poland.

## Author Contributions

MS was responsible for study design, data collection, data analysis, and writing of the manuscript. BT, ZA, and BS contributed to data collection and data analysis. AL contributed to study design, and writing of the manuscript. All authors were involved in writing the paper and approved the submitted final versions.

### Conflict of Interest Statement

The authors declare that the research was conducted in the absence of any commercial or financial relationships that could be construed as a potential conflict of interest.

## Abbreviations

CRP: C reactive protein
ESR: erythrocyte sedimentation rate
FNAB: fine needle aspiration biopsy
FT4: free thyroxine
HLA: human leukocyte antigens
MHC: major histocompatibility complex
RAIU: radioiodine uptake
SAT: subacute thyroiditis
TSH: thyrotropin
US: ultrasound.
